# Effect of Cetylpyridinium Chloride (CPC) on Colony Formation of Common Nontuberculous Mycobacteria

**DOI:** 10.3390/pathogens7040079

**Published:** 2018-10-05

**Authors:** Myra D. Williams, Joseph O. Falkinham

**Affiliations:** Department of Biological Sciences, Virginia Tech, Blacksburg, VA 24061, USA; mywillia@vt.edu

**Keywords:** nontuberculous mycobacteria, cetylpyridinium chloride, decontamination

## Abstract

Cetylpyridinium chloride (CPC) is widely used to decontaminate water samples for the cultivation of nontuberculous mycobacteria (NTM). The rationale for using CPC is that it kills more non mycobacteria than NTM and thereby prevents the outgrowth and detection of mycobacterial colonies on solid media. The few CPC-susceptibility measurements that have been published, suggest that CPC-decontamination does kill significant numbers of NTM. We confirm that observation here and further demonstrate that CPC-susceptibility varied significantly by one log between representative NTM species and between strains of the same species. CPC-susceptibility was the same for cells collected from cultures or water-acclimated (*P* = 0.6485, *T*-test) and CPC-susceptibility was relatively similar over the range of commonly employed CPC dosages. We conclude that use of CPC as decontaminating agent may lead to failure to recover an NTM isolate and considerable underestimates of NTM numbers.

## 1. Introduction

A major problem of mycobacterial detection and enumeration by cultivation is the slow growth of *Mycobacterium* spp. and the resulting outgrowth and detection of mycobacterial colonies by other, faster growing, microorganisms. This is especially true when one is attempting to isolate mycobacterial isolates from water, biofilm, and soil samples for DNA fingerprinting. The presence of other microorganisms is expected because all those types of environmental samples are rich in non-mycobacterial, faster growing microorganisms. Accordingly, there has been a need to decontaminate samples prior to cultivation. A wide variety of agents, including 4% NaOH, 5% oxalic acid, and 0.1% cetylpyridinium chloride (CPC), have been used as decontaminating agents with success [1,2,3,4,5,6,7]. As shown by Radomski et al. [4], fewer mycobacterial cells are killed following exposure to 0.1% CPC compared to non-mycobacterial cells, leading to increased opportunity to detect mycobacterial colonies. CPC has the additional valuable property of detergent activity, liquefying sputum [1] and disrupting biofilms to allow the release of mycobacterial cells from the biofilm extracellular matrix.

In our attempts to identify household sources of the nontuberculous mycobacteria (NTM), we have often found that decontamination is required for soil, drinking water, and pipe biofilm samples [5]. To isolate NTM from such samples, we have used CPC at a dosage of 0.1% for 30 min [5]. That choice of concentration was based on the report that 0.1% CPC resulted in killing 1–2 logs of cells of strains of *Mycobacterium chelonae* or *Mycobacterium avium*, but killed 2–3 logs of non-mycobacterial cells [4]. Although higher and lower concentrations of CPC have been employed for decontamination [1,6,7,8], we chose 0.1% CPC to avoid higher levels of mycobacterial killing without the possible overgrowth of non-mycobacterial colonies. Although NTM colonies have been recovered from CPC-exposed samples, we have assumed that CPC-susceptibility of all NTM strains is similar. However, there is the possibility that the data of Radomski et al. [4] did not reflect CPC-susceptibility of other NTM species or strains and that of NTM cells acclimated to water, their natural habitat. Possibly, CPC-decontamination could: (1) reduce the number of NTM colony-forming units, and (2) present a false picture of the NTM population as NTM species may not be uniformly susceptible to CPC. As this laboratory is principally focused on isolation of NTM from premise plumbing samples, we needed to measure the survival of NTM cells to CPC whether grown in laboratory medium or following water-acclimation. Our hypothesis was that culture-medium-grown NTM cells were more susceptible to CPC compared to water-acclimated cells. To test that, the water-acclimated cells were employed as surrogates for NTM from natural and human engineered water systems and compared to culture-grown cells.

## 2. Results

### 2.1. Effect of 0.1% CPC Exposure for 30 Minutes on CFU/mL

Exposure to 0.1% CPC for 30 min resulted in a wide range of survival values (Table 1). Statistical analysis comparing the colony-forming units (CFU)/mL values of CPC-exposed and the control suspensions for the individual strains, documented that the differences were significant (*T*-test) except for those instances where survival after CPC exposure was quite high (Table 1). However, there was no significant difference between the per cent survival of culture-grown and water-acclimated cells (*P* = 0.6485, *T*-test) for all the strains except *M. abscessus* strains PS-W-4-1 and MHS-Sw-7-1.

No single survival value could be assigned to these representative *Mycobacterium* species and percent survivals were quite different for the different isolates of strains of the same species; namely *M. avium, M. chimaera*, and *M. abscessus* (Table 1). Colony type morphology evidently is a determinant of CPC-susceptibility as the transparent variant of *M. avium* strain Va14, Va14 (T) is considerably more tolerant of CPC than its interconvertible and isogenic opaque variant; Va14 (O) (Table 1).

### 2.2. Effect of Different CPC Dosages on CFU/mL

Within the range of CPC concentrations that have been used in studies from the literature (i.e., 0.05–0.20%) and exposure durations (i.e., 15–120 min) examined [1,3,4,5,6,7,8], there were little differences in NTM survival. In fact, the lowest CPC dosage (0.05% for 30 min) led to similar survival values as cells exposed to 0.10% CPC for 30 min or 0.10% CPC for 60 min. (Table 2). Exposure to 0.2% CPC for 30 min reduced survival by 55% compared to exposure to 0.1% CPC for 30 min (Table 2).

## 3. Discussion

Cells of a variety of NTM species, specifically *M. avium*, *M. intracellulare*, *M. chimaera* and *M. abscessus* did not exhibit the same susceptibility to 0.1% CPC exposure for 30 min. Even within a single species or between strains, susceptibility was quite different (Table 1). That means one cannot estimate the actual numbers of NTM cells in environmental samples based on applying a correction-factor to colonies recovered. The major factors responsible for the differences were species and strain, not growth conditions or water-acclimation. The fact that opaque variants were considerably more susceptible to CPC was not unexpected, but the wide difference in CPC-susceptibility of strains of the same species (e.g., *M. chimaera*), was not anticipated (Table 1). The differences between the transparent (T) and opaque (O) colony types of *M. avium* strain Va14 is consistent with observations that transparent variants are more hydrophobic and antibiotic- and metal-tolerant, compared to their opaque variants [9,10].

That CPC-susceptibility was relatively constant across a range of concentrations (i.e., 0.05–0.20%, Table 2) and exposure durations (i.e., 15–60 min, Table 2), suggests there is little room to increase NTM survival by alteration of CPC-dosage. Thus, we conclude that whenever CPC-decontamination is employed to prevent overgrowth and isolate NTM colonies, the resulting colony counts are underestimates of the true NTM numbers in water or biofilm samples.

Laboratory measurements of NTM colony counts show wide variation in part due to aggregation of NTM cells, driven by surface hydrophobicity [10,11]. Aggregation leads to large variations in colony counts due to two factors. First, aggregates protect cells from CPC exposure in much the same way as biofilms protect cells from decontaminating agents. Second, when suspensions containing aggregates are spread on agar medium, spreading may or may not fully dissociate cells in the aggregate. In our experience, aggregates of medium-grown or water-acclimated suspensions of NTM cells usually contain from between 10–100 cells [12]. Thus, vigorous spreading to dryness on agar media can increase colony counts of aggregated cell suspensions by factors of 10–100-fold. In the absence of vigorous spreading, an aggregate may remain intact and yield a single colony. Aggregates are also subject to dispersal due to the detergent action of CPC. To reduce the effect of aggregation, suspensions must be vortexed at the highest setting for 60 sec and the liquid suspensions spread to dryness on relatively dry M7H10 agar plates (i.e., 3 days old) as was performed here.

There are few other studies using CPC-decontamination where NTM survival has been measured. Generally, a variety of CPC concentrations have been used [1,3,4,5,6,7,8] and NTM isolated, but few survival values have not been provided. In the study cited above, a decontamination regimen of 0.1% CPC exposure for 30 min followed by neutralization, killed 2–3 logs of non-mycobacterial cells, but only 1–2 logs of *Mycobacterium chelonae* cells [4]. That information and presentation in parallel bar graphs elegantly documented the value of decontamination; killing is higher for non-mycobacterial cells allowing the detection, isolation, and identification of NTM from environmental samples. Although one-log killing was also observed here for some of the NTM cells, killing of some NTM strains was far higher (Table 1).

Decontamination, by whatever means, to detect and recover NTM colonies from environmental samples, significantly reduces the sensitivity of detection. Generally, 100–1,000 NTM cells per mL are found in premise plumbing water samples [5]. Unless very large samples are collected, some NTM cells will not be detected, not because they are absent, but because the cells were lost as colony-forming units by decontamination. Clearly, that limits our ability to identify NTM sources and to perform risk-assessment. As colonies are required (at present) for DNA-fingerprinting to link patient and environmental isolates, either alternative decontamination regimens are needed such as those using Tween-80 or N-acetyl-cysteine [13] that may be gentler on NTM or selective media need to be developed for NTM [14].

## 4. Materials and Methods

### 4.1. Mycobacterium *spp.* Strains

The following strains were employed in this study, chosen on the basis that they represent the species and types most often recovered from patients and epidemiologically-linked water or biofilm samples in the United States. The strains include: *Mycobacterium avium* strain A5, a plasmid-free patient isolate [15]; *M. avium* strain Va14 (T) a transparent colony-type aerosol isolate collected by the James River in Richmond, Virginia [12]; *M. avium* strain Va14 (O), a spontaneous opaque colony-type variant isolated from a culture of *M. avium* strain Va14 (T); *Mycobacterium intracellulare* strain TMC 1406^T^ from the Trudeau Memorial Collection and type strain for *M. intracellulare*; *Mycobacterium chimaera* strain MA3323, a patient isolate; *M. chimaera* strain NC-W-2-1, a water isolate recovered from a Sorin 3T heater cooler; *Mycobacterium abscessus* strains AAy-P-1, an isolate from a patient residing in the San Francisco Bay area; *M. abscessus* strain PS-W-4-1, a plumbing biofilm isolate from a Florida patient’s home, *M. abscessus* strain MHS-Sw-2-1 isolated from a plumbing biofilm sample collected from a Florida hospital, and *M. abscessus* strains 12-9-Sw-B-2 and 12-45-Sw-A-1 isolated from plumbing biofilms from two different homes in Hawai’i [16].

### 4.2. Growth and Collection of Mycobacterium *spp.* Strains

Strains were grown from single colonies on Middlebrook 7H10 agar medium (Becton-Dickenson, Sparks, MD) containing 0.5% (vol/vol) glycerol and 10% (vol/vol) oleic acid-albumin inoculated into 2 mL of M7H9 broth (Becton–Dickenson, Sparks, MD) containing 0.5% (vol/vol) glycerol and 10% (vol/vol) in a 16-125 mm screw capped tube and incubated for 7 days to an absorbance 540 nm of 0.300 to avoid aggregation that occurs in mycobacterial cultures at absorbances (540 nm) above 0.500. That culture was sampled and if it was not contaminated and colonies were of the same morphology as the colony picked (>95%), 1 mL was used to inoculate 50 mL M7H9 broth in a 500 mL nephalometer flask at 37 °C with aeration (120 rpm) for 7 days to mid-log phase.

### 4.3. Water Acclimation

A portion (5 mL) of the culture-grown cells was collected by centrifugation (5000× *g* for 20 min) and suspended in an equal volume of sterile Blacksburg tap water. Those cells were washed twice in sterile Blacksburg tap water and re-suspended in 5 mL of sterile Blacksburg tap water. The twice-washed cells were incubated for 7 days at room temperature with aeration (120 rpm) to acclimate the M7H9-broth grown cells to water.

### 4.4. Measurement of the Effect of CPC on Colony-Forming Units (CFU) /mL

Culture-grown or water-acclimated suspensions were either not treated (control) or exposed to cetylpyridinium chloride (CPC, Sigma-Aldrich, St. Louis, MO) added to a final concentration of between 0.05–0.20% and incubated for different time periods (15–120 min). Following incubation, colony counts of all *Mycobacterium* spp. suspensions were measured by spreading 0.1 mL of undiluted or diluted from untreated or CPC-containing suspensions on M7H10 agar medium in triplicate. Immediate spreading on M7H10 agar leads to inactivation of CPC’s detergent activity by albumin. Colonies were counted after 14 days incubation at 37 °C. Each experiment reported in the tables was repeated at least twice. Two separate experiments were performed to: (1) measure the effect on CFU/mL of the culture-grown and water-acclimated suspensions exposed to the lab’s standard CPC exposure (0.1% CPC for 30 min) and (2) measure the effect of different CPC concentrations (0.05–0.20%) and durations of exposure (15–120 min) on CFU/mL of the culture-grown and water-acclimated suspensions. The data are reported as the number of CFU before and after CPC-exposure expressed as a percent.

### 4.5. Statistical Analysis

Comparisons between CFU/mL values for control and CPC-exposed suspensions of culture-grown and water-acclimated cells were performed with Student’s *t*-test using Instat® Version 3.0 (GraphPad Software, Inc. San Diego, CA, USA).

## Figures and Tables

**Table 1:** Susceptibility of culture-grown and water-acclimated *Mycobacterium spp.* to 0.1% CPC for 30 min.

Strain	Percent Survival ^a^ (*T*-test) ^b^Culture-Grown	Percent Survival ^a^ (*T*-test) ^b^Water-Acclimated
*M. avium A5*	21.7% (0.0049)	20.6% (0.0125)
*M. avium Va14 (T)*	64.7% (0.3176)	81.3% (0.0748)
*M. avium Va14 (O)*	1.6% (0.0088)	3.8% (0.0017)
*M. intracellulare 1406^T^*	13.4% (0.0427)	23.1% (0.0009)
*M. chimaera MA3323*	21.2% (0.0029)	26.3% (0.0061)
*M. chimaera NC-W-2.1*	64.0% (0.0158)	68.9% (0.0132)
*M. abscessus AAy-P-1*	34.2% (0.0076)	24.0% (0.0014)
*M. abscessus PS-W-4-1*	62.6% (0.1057)	23.9% (0.0014)
*M. abscessus MHS-Sw-7-1*	33.5% (0.0008)	6.6% (< 0.0001)
*M. abscessus 12-9-Sw-B-2*	5.9% (< 0.0001)	6.4% (0.0041)
*M. abscessus 12-45-Sw-A-1*	25.0% (0.0041)	36.7% (0.0242)

^a^ CFU/mL of CPC-exposed divided by CFU/mL of control, expressed as percent. **^b^** *T*-test *P*-value for CFU/mL differences in control and CPC-exposed suspensions.

**Table 2:** Effect of CPC dosage on colony count of *M. avium* strain A5.

CPC (% wt/vol)	Exposure (min)	Per Cent Survival ^a^Culture-Grown
0 (Control)	0 (Control)	100%
0.05%	30 min	16.7 ± 2.8
0.10%	15 min	15.4 ± 2.7
0.10%	30 min	16.4 ± 5.0
0.10%	60 min	12.2 ± 3.7
0.20%	30 min	9.0 ± 0.1

^a^ CFU/mL of CPC-exposed divided by CFU/mL of control, expressed as percent.
